# Influence of Selective Laser Trabeculoplasty (SLT) on the iStent inject® outcomes

**DOI:** 10.1186/s12886-020-01723-3

**Published:** 2020-11-19

**Authors:** Anna-Karina B. Maier, Parisa Arani, Milena Pahlitzsch, Anja-Maria Davids, Daniel Pilger, Matthias K. J. Klamann, Sibylle Winterhalter

**Affiliations:** Department of Ophthalmology, Campus Virchow- Klinikum, Charité – University Medicine Berlin, corporate member of Freie Universität Berlin, Humboldt- Universität zu Berlin and Berlin Institute of Health, Berlin, Germany

**Keywords:** SLT, iStent inject

## Abstract

**Background:**

To evaluate the influence of Selective Laser Trabeculoplasty (SLT) on iStent inject® outcomes in open-angle glaucoma (OAG).

**Methods:**

In this retrospective comparative cohort outcome study, 66 patients who were treated with two iStent inject® devices were included. Patients were divided into two subgroups consisting of patients without SLT treatment prior to surgery and patients who had been treated previously with 360° SLT but without sufficient response. Outcome measures included intraocular pressure (IOP) and number of antiglaucoma medications after 6 weeks with three, six, 12, and 24 month follow-ups.

**Results:**

Mean preoperative IOP decreased from 20.4 ± 5.3 mmHg to 14.8 ± 3.0 mmHg for patients without SLT treatment prior to surgery (*p* = 0.001) and from 19.2 ± 4.5 mmHg to 14.0 ± 1.6 mmHg for patients with insufficient response to 360° SLT treatment (*p* = 0.027) at 12 months after iStent inject® implantation. No significant difference was found between the two groups (*p* >  0.05). The number of antiglaucoma medications did not change in both groups (*p* >  0.05) and showed no significant difference between the two groups (*p* >  0.05).

**Conclusion:**

Prior SLT treatment seems to have no negative influence on the IOP lowering-effect of iStent inject® implantation in patients with OAG. It is therefore an appropriate incremental procedure with no exclusion criterion for an iStent inject® implantation.

## Background

The treatment of Primary Open Angle Glaucoma (POAG) and secondary Glaucoma like Pseudoexfoliation Glaucoma (XFG) or Pigment dispersion Glaucoma remains a challenge despite several treatment methods. The primary focus of these methods is treating the elevated intraocular pressure (IOP), because an elevated IOP is a major risk factor for the development and progression of glaucoma. Medical treatment, laser treatment and surgery are the available methods to reduce IOP.

Selective Laser Trabeculoplasty (SLT) is one possibility of laser treatment. The method uses a 532 nm Nd:YAG laser to target the pigmented cells of the trabecular meshwork [1]. Several studies demonstrated that SLT is an efficient and safe method to reduce sustainably the IOP in glaucoma patients [1–13], although the mechanism remains uncertain. Three possible involved mechanisms are discussed: dislodging of trabecular cells, mechanical distension of Schlemm’s canal, stimulation of cellular production, and turnover of extracellular matrix [14]. In contrast to the argon laser trabeculoplasty (ALT), histologic and ultrastructural studies found less extensive damage and no coagulative effects on the trabecular meshwork after SLT [12, 15, 16]. Nonetheless, mechanical damages can still be observed because SLT produces disruption of trabecular beams, accumulation of cellular debris, fragmentation and sloughing of endothelial cells [15] and results in an increased trabecular meshwork monocyte recruitment as a result of increased chemokine production [12, 17]. Additionally, in-vitro-experiments showed an increase in pro-inflammatory cytokine expression [12, 18].

Micro-invasive glaucoma surgery (MIGS) is used more and more often and can easily be combined with microincision cataract surgery. The iStent inject® (Glaukos Corporation, Laguna Hills, CA, USA) is one of the available MIGS and fulfils the criteria of MIGS: ab interno microincision, minimal trauma, efficacy, high safety profile and rapid recovery [19]. The target structure of performing this procedure is also the trabecular meshwork. As the trabecular meshwork is considered as the primary source of resistance to aqueous drainage in many glaucoma forms, the aim of the iStent inject® is lowering the IOP by bypassing the trabecular meshwork and using the natural physiological pathways behind it [20–23]. Trabectome is an alternative MIGS with the same intent as the iStent inject®. Prior SLT treatment seems to have no negative influence on combined clear cornea phacoemulsification and Trabectome outcomes in glaucoma patients [24], although it leads to an alteration of the trabecular meshwork. But Wimmer et al. showed, that ALT appears to increase the risk of bleb scarring in XFG patients after trabeculectomy because of increased levels of activated TGF-beta 2. In addition, Khalili et al. demonstrated a significant lower success rate in terms of normalization of IOP after trabeculectomy in patients with prior argon laser trabeculoplasty [25, 26]. Since SLT may lead to mechanical damage and results in chemokine production, SLT may affect a subsequent glaucoma surgery and its success.

The impact of SLT treatment and the associated alteration of the trabecular meshwork previously performed to iStent inject® implantations remains unclear. Our hypothesis is that the prior SLT could decrease the treatment effect of iStent inject® implantations. Therefore, this study examined the influence of SLT treatment on iStent inject® outcomes in open-angle glaucoma (OAG).

## Methods

In this retrospective, comparative, cohort, outcome study between June 2014 and February 2016, we included eyes from patients that underwent MIGS with implantation of two iStent inject® devices at the Department of Ophthalmology, Charité – Universitätsmedizin Berlin. All patients diagnosed with moderate OAG (including primary and pseudoexfoliative glaucoma) who underwent MIGS with implantation of two iStent inject® devices were included into this study. Cases with missing follow-up data, with previous incisional procedures (trabeculectomy, tube shunts, Trabectome, cyclophotocoagulation) and implantations combined with cataract surgery were excluded. Only data of the right eye were included in patients who underwent implantation of iStent inject® devices on both eyes. If a secondary glaucomatous surgery during the follow-up time was necessary data were not analysed anymore after the time point of secondary glaucomatous surgery. Ethic approval had been given by the Ethikkommission, Charité – Universitätsmedizin Berlin, EA4/047/20. For this type of study formal consent is not required because it is a retrospective, single-center study and the Ethikkommission, Charité – Universitätsmedizin Berlin approved the waiver of consent. This study adhered to the ethical standards of the Declaration of Helsinki. An informed written consent was provided for surgery. The following was performed for each patient: a complete ophthalmological examination – including a medical history review – best corrected visual acuity (BCVA) measurement tested with a Snellen chart, slit-lamp examination, IOP measurement using Goldmann’s applanation tonometry, gonioscopy, dilated fundus examination, stereoscopic photographs of the optic disc, a baseline bilateral standard automated perimetry threshold visual test using the 30–2 Tendency-Oriented Perimetry (TOP) programme (Octopus, Haag-Streit), and a baseline peripapillary retinal nerve fibre layer (RNFL) thickness measurement by Spectralis optical coherence tomography (OCT) (Spectralis OCT, Heidelberg Engineering GmbH, Heidelberg, Germany).

Mild- or early stage open angle glaucoma and moderate open angle glaucoma were defined as described by Gonnermann et al. [23]. Two independent observers categorised the visual field status of all patients before surgery as mild, moderate or advanced based on the 30–2 Tendency-Oriented Perimetry programme. In cases of disagreement, the visual field status was judged by a third senior glaucoma specialist (MK). In this study we included only patients with mild and moderate visual field defects. Average RNFL thickness was documented for mild and moderate stage, defining the open angle glaucoma.

Drawing from routine questionnaires given to all patients prior to examination, it is to the author’s best knowledge that all patients were free from other ocular diseases apart from glaucoma and cataract.

MIGS with implantation of two iStent inject® devices was performed in patients with OAG because of two reasons. The first reason was an insufficient IOP despite well tolerated local antiglaucomatous therapy and the preference for a minimally invasive procedure. The second reason was an insufficient IOP without local therapy. In these patients the minimally procedure was chosen to avoid a local antiglaucomatous therapy. In all other cases, where the local therapy was not well tolerated or a low target pressure was needed because of an advanced glaucoma, an alternative procedure (trabeculectomy or glaucoma drainage device) was performed and patients were not included. All included patients were divided into two subgroups. These included patients without previous SLT treatment (group A) and patients who had insufficient or no longer sufficient IOP reduction after 360° SLT treatment with a minimum of 3 months prior to surgery (group B) – the Trabeculas SLT (A.R.C. Laser, Nuernberg, Germany) using 95–105 spots applied to the trabecular meshwork. The evaluation of success rates (IOP < 18 mmHg) after SLT procedure could be shown to be 74.5% after 3 years (data submitted for publication). The main reasons for initially performed SLT before following iStent inject® implantation was a barely not achieved target pressure (2–4 mmHg above target pressure) with or without local antiglaucomatous therapy or patients preference to avoid initially a surgery. Insufficient or no longer sufficient IOP reduction after SLT treatment was defined as an IOP above the target pressure, needing an increase of the antiglaucoma medication in routine examination with a minimum of 3 months after SLT in the study eye. The target pressure was individualized based on the factors recommended by the European Glaucoma Society including stage of glaucoma, IOP before treatment, age and life expectancy, rate of progression and presence of risk factors for progression.

Goldmann applanation tonometry was performed measuring IOP and the topical antiglaucoma medications applied were noted 1 day preoperatively, and then on a 1 day, six-week, three-, six-, 12-, 24- month frequency postoperatively.

### Surgical technique

All procedures were performed by three experienced surgeons (MK, JG, NT) using the same surgical protocol under topical anaesthesia.

The implantation of two iStent inject® devices was performed as published before after an ophthalmic viscosurgical substance was injected into the anterior chamber for stability [22, 23]. Per protocol, the iStents were inserted nasally under gonioscopic control through the trabecular meshwork into Schlemm’s canal, separated by approximately two clock hours.

Standard postoperative treatment included a topical combination of steroids and antibiotics. Following the surgery, therapy was reduced over a period of 4 weeks. Antiglaucoma medication was used by patients as needed.

### Statistical methods

Statistical analysis was performed using IBM SPSS statistics 19 (SPSS Software, Munich, Germany). A sample size calculation was based on the assumption of a mean postoperative IOP 11.83 ± 2.21 mmHg based on the available data in the literature and a distribution of 4:1 [24]. At a power of 80% and an alpha level of 5%, we estimated that a group size of 60 patients would allow to detect a difference of 2 mmHg. A post-hoc power analysis revealed that we could find a difference of 3 mmHg at an alpha of 5% and a power of 80%.

Descriptive statistics were expressed as mean ± standard deviation (SD) and minimum and maximum. Normality was tested for all outcome measures and the appropriate statistical test was used. We used nonparametric tests (Wilcoxon signed-rank test, Mann-Whitney-U test). Kaplan-Meier survival analysis and the log-rank test were used to analyze the second surgery free survival incidence. To explore independent risk factors for IOP, we entered preoperative parameters (patient’s age, status of lens, POAG versus XFG, SLT treatment prior to surgery or not) into a linear regress model. Differences were considered statistically significant when *p*-values were less than 0.05.

## Results

Between June 2014 and February 2016, 193 eyes of 170 patients diagnosed with moderate OAG (including primary and pseudoexfoliative glaucoma) who underwent MIGS with implantation of two iStent inject® devices were screened for the study. Fifteen eyes with missing follow-up data, 23 eyes because of the second eye of a patient, 74 eyes with implantations combined with cataract surgery and 15 eyes with previous incisional procedures (trabeculectomy, tube shunts, Trabectome, cyclophotocoagulation) were excluded. In total, sixty-six eyes of 66 Caucasian patients (35 females, 31 males; mean age 73.1 ± 11.7 years) with moderate OAG (POAG *n* = 45 and XFG *n* = 21) were included in the study. In all cases, two iStent inject® devices were implanted. The average follow-up time was 539 ± 285 days.

**Fig. 1:**
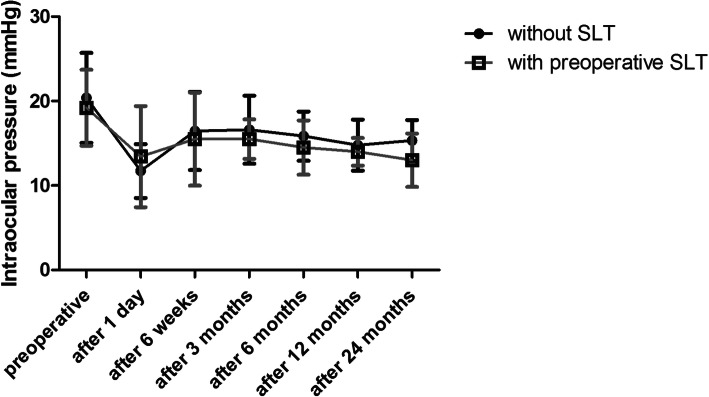
Influence of Selective Laser Trabeculoplasty (SLT) on the iStent inject® outcomes. Date of IOP (ordinate [mmHg]) over time (abscissa [time after surgery]) from eyes classified as OAG, given separately for eyes without SLT treatment prior to surgery (black) and eyes who had insufficient response to 360° SLT treatment previous to surgery (grey). Date of IOP over time are presented as mean value and standard deviation (SD) preoperatively and at postoperative 1 day, 6 weeks, 3, 6, 12 and 24 months after surgery

**Fig. 2:**
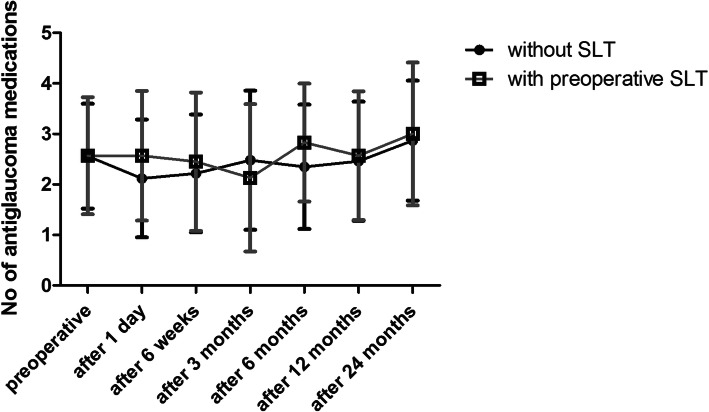
Influence of Selective Laser Trabeculoplasty (SLT) on the iStent inject® outcomes. Number of topical medications (ordinate [n]) over time (abscissa [time after surgery]) from eyes classified as OAG, given separately for eyes without SLT treatment prior to surgery (black) and eyes who had insufficient response to 360° SLT treatment previous to surgery (grey). Data of number of antiglaucoma medications are presented as mean value and standard deviation preoperatively and at postoperative 1 day, 6 weeks, 3, 6, 12 and 24 months after surgery

Table 1 presents the preoperative characteristics. Figure 1 shows the IOP measurements and Fig. 2 the change in number of antiglaucoma medication over time for both groups.

**Table 1:** Preoperative characteristics

	OAG (*n* = 66)		
	Without prior SLT to surgery A	With SLT treatment previous to surgery B	*p*-values
Intraocular pressure (IOP)			
- Mean (SD)	20.38 (5.33)	19.21 (4.49)	*p* = 0.539
- Range	15–38	15–31	
No. of medications			
- Mean (SD)	2.56 (1.04)	2.57 (1.16)	*p* = 0.915
- Range	0–4	0–4	
- No. on 0 meds (%)	1 (1.9%)	1 (7.1%)	
- No. on 1 meds (%)	9 (17.3%)	1 (7.1%)	
- No. on 2 meds (%)	11 (21.2%)	4 (28.6%)	
- No. on 3 meds (%)	22 (42.3%)	5 (35.7%)	
- No. on 4 meds (%)	9 (17.3%)	3 (21.4%)	
Cup-to-Disc ratio			
- No. ≤ 0.3 (%)	0 (0%)	0 (0%)	*p* = 0.508
- No. > 0.3 ≤ 0.5 (%)	6 (11.5%)	2 (14.3%)	
- No. > 0.5 ≤ 0.8 (%)	25 (48.1%)	6 (42.9%)	
- No. > 0.8 (%)	21 (40.4%)	6 (42.9%)	
Best-corrected visual acuity			
- ≥ 20/40 (%)	39 (75.0%)	9 (64.3%)	*p* = 0.259
- 20/50 to 20/100 (%)	7 (13.5%)	1 (7.1%)	
- ≤ 20/200 (%)	6 (11.5%)	4 (28.6%)	
Visual Field			
- Mild (%)	12 (23.1%)	5 (35.7%)	*p* = 0.337
- Moderate (%)	40 (76.9%)	9 (64.3%)	
- Advanced (%)	0 (0%)	0 (0%)	
Average RNFL thickness in μm			
- Mild - Mean (SD)	86.5 (12.1)	88.4 (3.8)	*p* = 0.897
- Moderate - Mean (SD)	68.1 (18.0)	61.4 (11.6)

A significant decrease was present in postoperative IOP compared to preoperative IOP at any time point (*p* < 0.001 after 12 months, *p* = 0.001 after 24 months). At 12 months, the average decrease in group A was 19.0% ± 20.7% (*p* = 0.001) and in group B 19.8% ± 11.8% without significant differences between the two groups (*p* = 0.981). Similarly, when considering absolute IOP values, no significant differences were noted during the entire follow-up period (preoperative *p* = 0.538, *n* = 66, after 1 day *p* = 0.720, *n* = 63, after 6 weeks *p* = 0.329, *n* = 55, after 3 months *p* = 0.364, *n* = 32, after 6 months *p* = 0.448, *n* = 32, after 12 months *p* = 0.633, *n* = 31 and after 24 months *p* = 0.171, *n* = 19).

The number of antiglaucoma medications did not change in both groups (*p* >  0.05) from 2.56 ± 1.04 in group A and 2.57 ± 1.16 in group B preoperatively to 2.46 ± 1.18 (*p* = 0.917) and 2.57 ± 1.27 (*p* = 0.317) at 12 months after surgery, respectively. Only 6 weeks after surgery the amount of topical medications was significantly decreased (*p* < 0.001). There was no significant difference in both groups during the entire follow-up period (preoperative *p* = 0.915, *n* = 66, after 1 day *p* = 0.160, *n* = 66, after 6 weeks *p* = 0.529, *n* = 56, after 3 months *p* = 0.532, *n* = 32, after 6 months *p* = 0.381, *n* = 32, after 12 months *p* = 0.825, *n* = 31 and after 24 months *p* = 0.712, *n* = 19).

Linear regression showed no association between preoperative parameters (patient’s age, status of lens, POAG versus XFG, SLT treatment prior to surgery or not) and postoperative IOP values after 12 and 24 months postoperatively (Tables 2 and 3).

**Table 2:** Data of linear regression for postoperative IOP values after 12 months

IOP after 12 months	Regression coefficient	95% confidence interval lower bound	95% confidence interval upper bound	*p*-value
Patient’s age	−0.101	−0.231	0.028	0.119
POAG versus XFG	0.931	−1.688	3.550	0.471
SLT treatment prior to surgery or not	−0.791	−3.258	1.676	0.516
Status of lens	−0.015	−3.511	3.481	0.993

**Table 3:** Data of linear regression for postoperative IOP values after 24 months

IOP after 24 months	Regression coefficient	95% confidence interval lower bound	95% confidence interval upper bound	*p*-value
Patient’s age	−0.005	−0.151	0.140	0.942
POAG versus XFG	−1.149	−4.861	2.562	0.517
SLT treatment prior to surgery or not	−2.142	−5.575	1.290	0.202
Status of lens	0.740	−3.364	4.845	0.705

Best-corrected visual acuity did not change significantly at any point in time. Additionally, there was no significant difference in BCVA between the two groups at any point in time during the entire follow-up (*p* >  0.05).

**Fig. 3:**
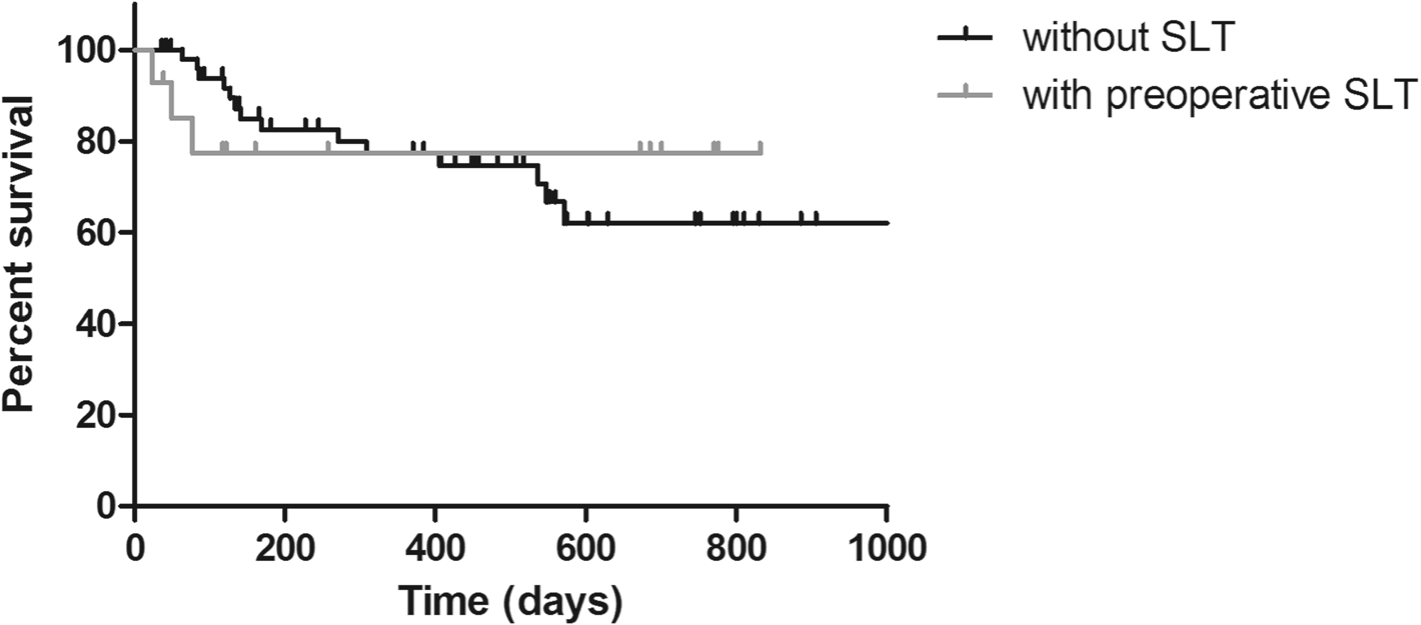
Influence of Selective Laser Trabeculoplasty (SLT) on the iStent inject® outcomes. Kaplan–Meier survival plot for eyes without SLT treatment prior to surgery (black) and eyes who had insufficient response to 360° SLT treatment previous to surgery (grey) with success defined as sufficient final intraocular pressure without additionally surgery. Secondary glaucomatous surgery had to be performed in 21.2% in group A and in 21.4% in group B due to insufficient IOP lowering-effect after MIGS (*p* = 0.788)

Apart from a reflux bleeding that occurred in 100% of patients, there were no severe intraoperative and postoperative complications including choroidal effusion, sustained hypotony, choroidal hemorrhage, or infection. The reflux bleeding resolved spontaneously itself. Secondary glaucomatous surgery had to be performed in 21.2% in group A and in 21.4% in group B due to insufficient IOP lowering-effect after MIGS (*p* = 0.788) (Fig. 3).

## Discussion

The study investigated the influence of previous SLT treatment on the outcomes of iStent inject® implantation in OAG. SLT and other glaucoma surgeries like trabectome, trabeculotomy and iStent inject® implantation have the same target: the trabecular meshwork. These procedures improve the outflow of aqueous humor. However, literary information about the influence of SLT on further glaucoma surgeries with the same target is sparse. The Trabectome Study Group showed data that previous laser trabeculoplasty did not affect the following Trabectome surgery negatively [27], however no differentiation between ALT, SLT, and micropulse diode laser trabeculoplasty modalities was made in the data collection. Furthermore, Klamann et al. demonstrated data that previous SLT treatment did not influence negatively combined clear cornea phacoemulsification and Trabectome outcomes in glaucoma patients [24]. Additionally, the effect of prior laser treatement (ALT, SLT) on the IOP lowering effect of trabectulectomy is not clear [25, 26, 28, 29].

According to the mean postoperative IOP, the mean number of antiglaucoma medications, and the number of eyes needing a secondary surgery to control IOP, we found no significant difference between patients without SLT treatment prior to iStent inject® implantation and patients who had insufficient or no longer sufficient response to 360° SLT treatment previous to surgery. Additionally, we found no correlation between the preoperative parameters, patient’s age, status of lens, POAG versus XFG, and especially SLT treatment prior to surgery or not, and postoperative IOP after 12 and 24 months postoperatively (*p* >  0.05). The iStent inject® implantation as a single procedure has shown to be effective in lowering IOP with minimal side effects as seen in our study [20–23]. Additionally, the effect in a lower IOP seems not to be influenced by a prior SLT treatment as hypothetically thought. The 360° SLT treatment alone is effective in lowering the IOP by improvement the outflow pathways of the whole trabecular meshwork in patients with XFG and PG [1–13]. In our study, if SLT treatment was insufficient or no longer sufficient to reduce IOP, a following iStent inject® implantation would lead to an IOP reduction by bypassing the trabecular meshwork – which is the major source of outflow resistance in open angle glaucoma. The previous treated pigmented cells and the associated alterations of the trabecular meshwork by SLT did not influence the IOP lowering-effect. Additionally, the number of eyes needing a secondary surgery to control IOP did not differ between SLT treated and non-treated groups. However, due to the study design, which included patients with an insufficient or no longer sufficient SLT, it has to be taken into account that the alterations of the trabecular meshwork by SLT in these patients might be less and this could be a reason for the missing influence. Based on our data, there was no significant group difference between patients with or without previous SLT. Further detailed studies that analyze inflammatory factors in the anterior segment and the alterations of the trabecular meshwork after SLT are necessary.

Nevertheless, an insufficient or a no longer sufficient SLT treatment should not be considered as an exclusion criterion for successful treatment by using iStent inject® implantation. Additionally, both interventions have the main advantage over standard filtrating procedures like trabeculectomy to increase an outflow facility along the natural pathway [20–23]. Although in some cases, an IOP lowering is not sufficient and other surgical procedures like filtrating or cyclodestructive procedures become necessary during follow-up sessions.

The main limitations of this study are the retrospective nature of the study design and the limited number of patients. Moreover, the follow-up time period of only 2 years is relatively short for glaucoma. Additionally, we included only patients with implantations of iStent inject® as single procedure and only patients with no other glaucoma surgery before. These might be selection bias. Therefore we cannot discuss the IOP lowering effect of iStent inject® implantations after SLT in more complicated cases and these factors may limit the generalizability of our findings to the entire population of patients with OAG. Additionally, the iStent inject® implanations were performed by 3 different surgeons in our study. Therefore, it is possible that surgeon depending differences influenced the results. To confirm the results of the present study, further prospective studies with a larger number of patients and a longer follow-up period are necessary.

## Conclusion

In conclusion, SLT is effective in lowering the IOP safely in a high number of glaucoma patients including POAG and XFG [1–13]. In this study, we did not find strong evidence for a negative effect of insufficient or no longer sufficient SLT prior to iStent inject® implantations. Nevertheless, SLT could have a negative effect on iStent inject® implantations but given our findings, we believe that such effect would be very small and possibly of no clinical importance. Therefore, SLT is an appropriate procedure prior to iStent inject® implantations with no exclusion criterion for an additional intervention on the trabecular meshwork.

## Data Availability

The datasets used and analysed during the current study are available from the corresponding author on reasonable request.

## Abbreviations

ALT: Argon Laser Trabeculoplasty
IOP: intraocular pressure
SLT: Selective Laser Trabeculoplasty
MIGS: Micro-invasive glaucoma surgery
OAG: open angle glaucoma
POAG: Primary Open Angle Glaucoma
XFG: Pseudoexfoliation Glaucoma
